# Antifragile Treatment for Efficient Chimerism of Induced Pluripotent Stem Cells Derived Hematopoietic Stem Cells

**DOI:** 10.1007/s12015-024-10828-x

**Published:** 2024-12-05

**Authors:** Daekee Kwon, Taewook Lee, Mijung Han, So-Woon Han, Kyung-Sun Kang

**Affiliations:** 1 Research Institute, Maru Therapeutics Co., Ltd., Office-706, Hangang-Misa-IS-BIZ Tower, Gyeonggi-do, 12925 South Korea; 2 Adult Stem Cell Research Center, College of Veterinary Medicine, Seoul National University, Seoul, 08826 South Korea

**Keywords:** Antioxidant, Conditioning, Engraftment, Hematopoietic Stem Cells Transplantation (HSCT), Induced Pluripotent Stem Cells Derived Hematopoietic Stem Cells (iHSC)

## Abstract

Engraftable hematopoietic stem cells (HSC) can be obtained from bone marrow, umbilical cord blood, and peripheral blood (PB). However, a major bottleneck in HSC transplantation is identifying an unrelated donor that completely matches the human leukocyte antigen type of the recipient. This issue can be resolved by producing patient-specific stem cells. The purpose of this study was to identify the conditions under which induced pluripotent stem cells (iPSC)-derived hematopoietic stem cells (iHSC) exhibit high efficiency. Because HSC are fragile and vulnerable to damage, this study was performed under the hypothesis that the engraftment rate could be increased by antifragile treatment. Antioxidant ginsenoside Rg1 was used to differentiate from iPSC to iHSC, and differentiated iHSC was intravenously injected into Balb/c nude mouse conditioned with diverse concentrations of busulfan. Engraftment was verified by the presence of human-specific markers in the PB at 2 and 8 weeks post iHSC transplantation. iHSC differentiated by incorporating 1 µM of Rg1 demonstrated high colony forming efficiency in vitro. Additionally, high efficiency engraftment occurred immediately after iHSC were transplanted into mice conditioned with high dose busulfan at a dosage of 125 mg/kg or higher. In this study, high-quality iHSC manufacturing and transplantation conditions capable of high efficiency engraftment in vivo were established. Hereafter, this method of producing HSC using patient-specific iPSC will become the fourth new source of HSC. Additionally, if gene-editing technology is applied, the scope of its application can be expanded to diverse infectious diseases.

**Graphical Abstract:**

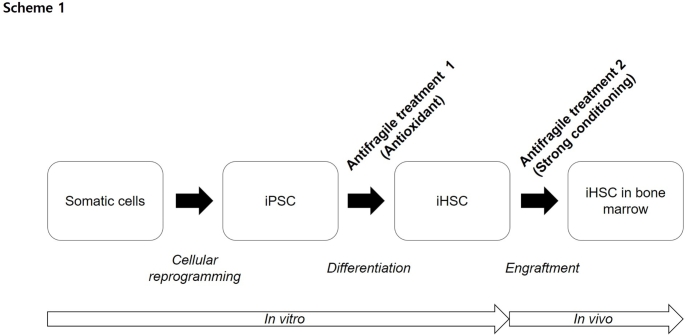

## Introduction

Human bone marrow (BM) is an important organ that produces approximately 500 billion blood cells daily. Hematopoietic stem cells transplantation (HSCT) is the final treatment for malignant blood tumors and autoimmune diseases caused by hematopoietic stem cells (HSC) in the BM. Currently, HSC capable of engraftment can be obtained from the BM, umbilical cord blood, or mobilized peripheral blood (PB). However, finding a HSC donor with a perfect recipient human leukocyte antigen (class I-A/B/C and class II-DR/DP/DQ) match remains the biggest bottleneck in HSCT [1]. Induced pluripotent stem cells (iPSC) derived hematopoietic stem cells (iHSC) can be a powerful means of overcoming the problem of matched donor shortage if they can be utilized for treatment [2]. Despite this, it is difficult to find reports on iHSC engrafted with high efficiency in vivo [3].

Research on the development of treatments using diverse cells differentiated from iPSC (e.g., neural stem cells, mesenchymal stem cells, T cells, and natural killer (NK) cells) is being actively conducted [4–7]. However, iHSC do not develop normally owing to their low engraftment rates. There are reports of iHSC differentiation with an increased engraftment rate through hematopoietic system-specific gene overexpression in the hemogenic endothelium (HE) [8]. Based on the general method of differentiating high-quality iPSC-derived cells with morphogen treatment alone, gene overexpression used for iHSC differentiation seems unlikely to be a fundamental solution.

The key to HSC engraftment is maintaining a high proportion of long-term repopulating hematopoietic stem cells (LT-HSC) in the HSC pool. A low reactive oxygen species (ROS) level is an essential condition for maintaining LT-HSC; when ROS levels rise, LT-HSC easily differentiate into short-term repopulating hematopoietic stem cells due to stress [9]. Thus, it was presumed that iHSC did not engraft well. However, we found that because HSC are very fragile compared to other cells, they receive irreversible damage from ROS during the arduous process of differentiation, resulting in a rapid decrease in functional HSC. If critical factors that cause damage to HSC during differentiation can be determined and minimized, LT-HSC can be minimally damaged and the engraftment rate will increase. Ginsenoside Rg1 is a good candidate for reducing the degree of damage to HSC caused by diverse external factors such as ROS [10, 11].

The busulfan/cyclophosphamide protocol is a standardized myeloablative conditioning regimen typically used before HSCT in adult patients. This protocol is a method of administering busulfan as intravenous (IV) 16 at 6 h intervals for 4 days, cyclophosphamide as IV 2 at 24 h intervals for 2 days, followed by HSCT 2 days later. During HSCT in an immunodeficient mouse model, it is common to use only busulfan, as the risk of immune rejection is lower than in humans. However, the conditioning protocols using busulfan are not standardized, and intraperitoneal (IP) administration of approximately 50–80 mg/kg in doses divided over 2–4 days is widely adopted. A non-standardized conditioning regimen may introduce bias into the derivation of iHSC engraftment results. Even in the case of chimeric antigen receptor-T cells therapeutics, efficacy varies significantly depending on the conditioning method [12]. Compared with other cells, the engraftment results of fragile iHSC can be greatly influenced by conditioning methods.

The first objective of this study was to obtain high-quality iHSC by reducing the damage caused by ROS during in vitro HSC differentiation (antifragile treatment I). The second objective was to optimize the in vivo busulfan conditioning regimen so that the iHSC produced could be engrafted into mouse BM with high efficiency (antifragile treatment II). Therefore, we explored the possibility of developing iHSC for use in translational research.

## Materials and Methods

### Animal Care

Male BALB/c nude mice (Central Lab Animal, KR) were used for in vivo engraftment assays. All animal experiments were performed in compliance with the guidelines approved by the Institutional Animal Care and Use Committee of Seoul National University (SNU-221228-4).

### HSC Differentiation from iPSC

All experiments using human cells were performed at the LMO Research Facility (Grade 2). This study was performed using dermal fibroblast-derived iPSC established by our research team [13]. iPSC was cultured in mTeSR plus medium (STEMCELL Technologies, US) in Matrigel (Corning, US)-coated dishes and subcultured by the mechanical method at 1-week intervals. For HSC differentiation, four iPSC clumps were seeded at low density (1 clump / cm^2^) in a Matrigel-coated 12-well dish. After attachment was induced for 2 days, differentiation was induced for 12 days by exchanging the medium with STEMdiff™ Hematopoietic Medium (STEMCELL Technologies). Each antioxidant was incorporated into the STEMdiff™ Hematopoietic Medium for 12 days to verify the antioxidant effects of ascorbic acid (Sigma, US), selenium (Sigma), Rg1 (Abcam, UK), and melatonin (Sigma). Previous studies reported additional concentrations of ascorbic acid, selenium, Rg1, and melatonin [14–16].

### Conditioning and in vivo Engraftment of iHSC

Busulfan (Sigma) was injected intraperitoneally into male BALB/c nude mice with IP injection for conditioning, and iHSC were injected intravenously 24 h later. Busulfan was diluted to a concentration of 10 mg/ml using dimethyl sulfoxide, and the target dose (20–200 mg / kg), according to the experimental design, was injected into the mouse as an IP (Table 2). Live iHSC (1 × 10^5^ / head) was suspended in 200 µl of PBS and then implanted as an IV in a conditioned mouse using an insulin syringe. Eight weeks post induced pluripotent stem cells derived hematopoietic stem cells transplantation (iHSCT), mice were sacrificed by CO_2_ gas injection, and the BM, peripheral blood mononuclear cells (PBMC), and spleen were sampled and used for human cell chimerism analysis.

### Analysis of Human Cell Chimerism

One to two weeks post iHSCT, a drop of PB was collected, and human cell chimerism analysis was performed by direct PCR using human/mouse-specific primers (Table 1) and Phusion Blood Direct PCR Master Mix (Thermo Fisher Scientific, US). PCR conditions were as follows: 98 ^o^C 5 min, 98 ^o^C 1 s, 60 ^o^C 5 s, 72 ^o^C 45 s (35 cycles), and 72 ^o^C 5 min. In the 8th week of iHSCT, PBMC, BM, and the spleen were sampled, and human cell chimerism was verified by FACS and PCR. BM mononuclear cells (MNC) were separated by flushing the inside of the left and right thighbones with PBS, and the spleen was completely lysed and used for analysis. PBMC were separated using the Ficoll gradient centrifugation method (STEMCELL Technologies). Human cell chimerism was computed using the following formula: Human cell chimerism (%) = [hCD45 (%) / (hCD45 (%) + mCD45 (%)) × 100]_experimental group_ − [hCD45 (%) / (hCD45 (%) + mCD45 (%)) × 100]_control group_.

**Table 1:** Used primer

Gene	Sequence (5` to 3`)	Size (bp)	Annealing temp. (^o^C)	Application
Human mtDNA I	F-CGAAAGGACAAGAGAAATAAGG	215	60	Genomic DNA PCR
	R-GTACAATGAGGAGTAGGAGGTTG			
Human mtDNA II	F-CTATCACCCTATTAACCACTCACG	912	60	
	R-GACTTGGGTTAATCGTGTGAC			
Mouse mtDNA	F-GATTTGAAGCCGCAGCATGATAC	1,506	60	
	R-GTCATAGGTGAACTCCATATAATGG			
Human CXCR4	F-TCCATTCCTTTGCCTCTTTT	1,068	55	
	R-TTAGCTGGAGTGAAAACTTG			
Human GAPDH	F-GTCAGTGGTGGACCTGACCT	349	60	
	R-TGCTGTAGCCAAATTCGTTG			

### In vitro Colony Forming Unit (CFU) Assay

To verify the multipotency of iHSC in vitro, 1 × 10^3^ iHSC were mixed with 2 ml MethoCult H4434 Classic Methylcellulose Medium (STEMCELL Technologies). The medium / cell mixture was placed in a 35 mm tissue culture plate (TCP) and incubated in an incubator at 37 ^o^C with 5% CO_2_ for 2 weeks. To maintain humidity, only DW was added, and the TCP with the lid removed was placed together. The total number and types of colonies formed were counted under a microscope at 40x magnification.

### Flow Cytometry

Flow cytometry was performed to analyze pluripotency markers, HSC markers, and human cell chimerism. Antibodies used include PE mouse anti-SSEA-4 (BD Biosciences, US), PE mouse anti-human TRA-1–60 (BD Biosciences), PE mouse anti-human TRA-1–81 (BD Biosciences), APC mouse anti-human CD34 (BD Biosciences), PE mouse anti-human CD45 (BD Biosciences), FITC rat anti-mouse CD45 (BD Biosciences), FITC mouse anti-human CD14 (BD Biosciences), and FITC mouse anti-human CD19 (BD Biosciences). The antibodies were diluted to 1/100 in FACS buffer (0.2% FBS in PBS). The antibodies were treated with live cells at 4 ^o^C for 30 min, washed twice, and FACS analysis was conducted. Flow cytometry was performed using a CytoFLEX (Beckman Coulter, US) or MACSQuant analyzer (Miltenyi Biotec, DE).

### ROS Quantification

In the case of iHSC differentiation, 0 / 1 / 10 µM of Rg1 was incorporated and differentiated, and intracellular ROS levels were quantified using 2` 7`-dichlorofluorescin diacetate (DCFDA) / H2DCFDA-Cellular ROS assay kit (Abcam). The cell permanent agent DCFDA was used to treat the iHSC for 30 min. Thereafter, fluorescence was measured using CytoFLEX (Beckman Coulter) (excitation/emission at 485/535 nm).

### Statistical Analysis

Data were analyzed by unpaired t-tests using the GraphPad Prism software (US). *p* < 0.01 or *p* < 0.05 was considered significant.

## Results

### Research Scheme

**Fig. 1:**
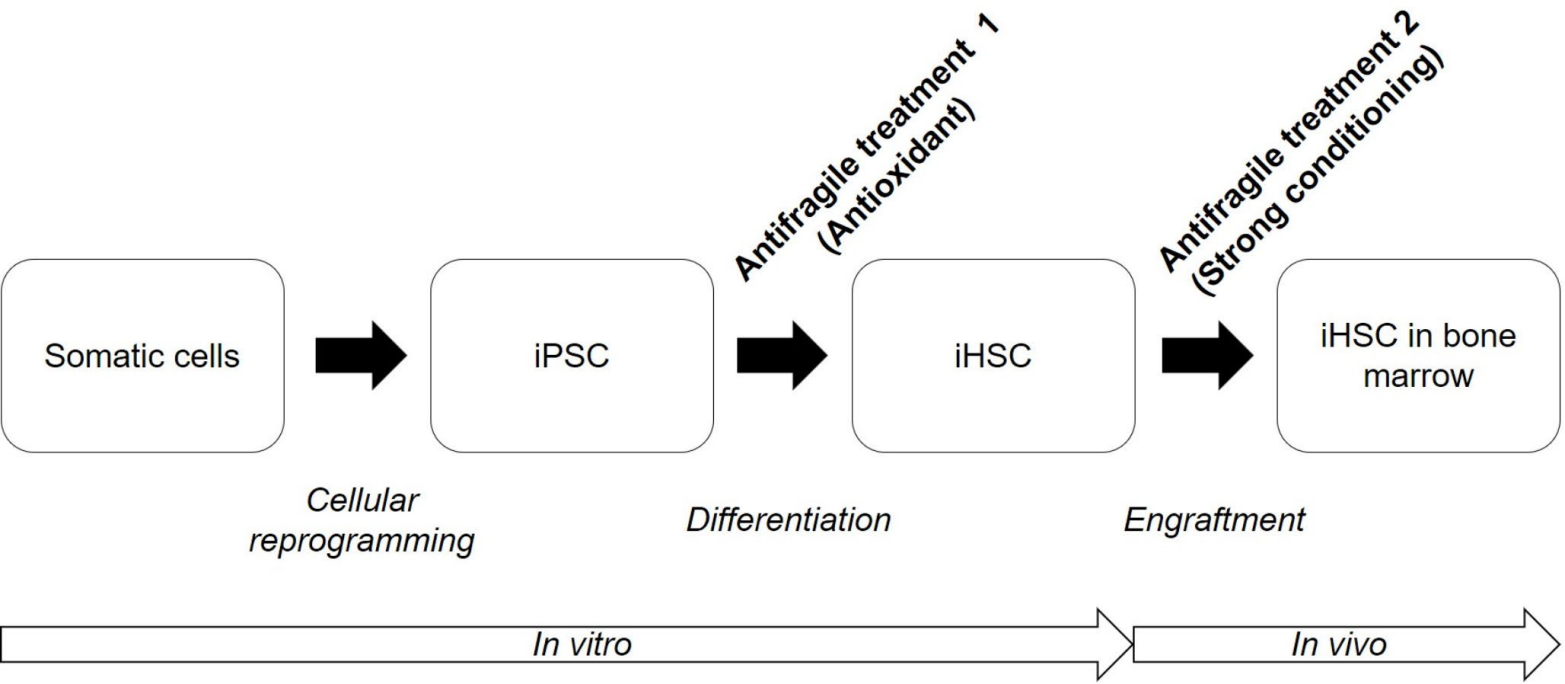
Study overview. Engraftable patient-specific iHSC can be generated. To this end, cell reprogramming, cell differentiation, and in vivo engraftment optimization technologies are convergently used. Engraftable iHSC are likely to be used for treating blood cancers, autoimmune diseases, and infectious diseases

Patient-specific engraftable HSC can be generated from iPSC. Dermal fibroblast-derived iPSC differentiate into iHSC. During iHSC differentiation, diverse antioxidants were screened. Among them, Rg1, which demonstrated the best effect, was selected. iHSC were implanted into nude mice conditioned with busulfan at diverse doses. Blood was collected in the second week post transplantation, and the presence of human cells was qualitatively evaluated using direct PCR. At the 8th week post transplantation, mice were sacrificed to isolate PBMC, BM, and the spleen, and human chimerism was quantified by FACS and PCR (Fig. 1).

### In vitro Generation of High Quality iHSC Using Antioxidant Rg1

**Fig. 2:**
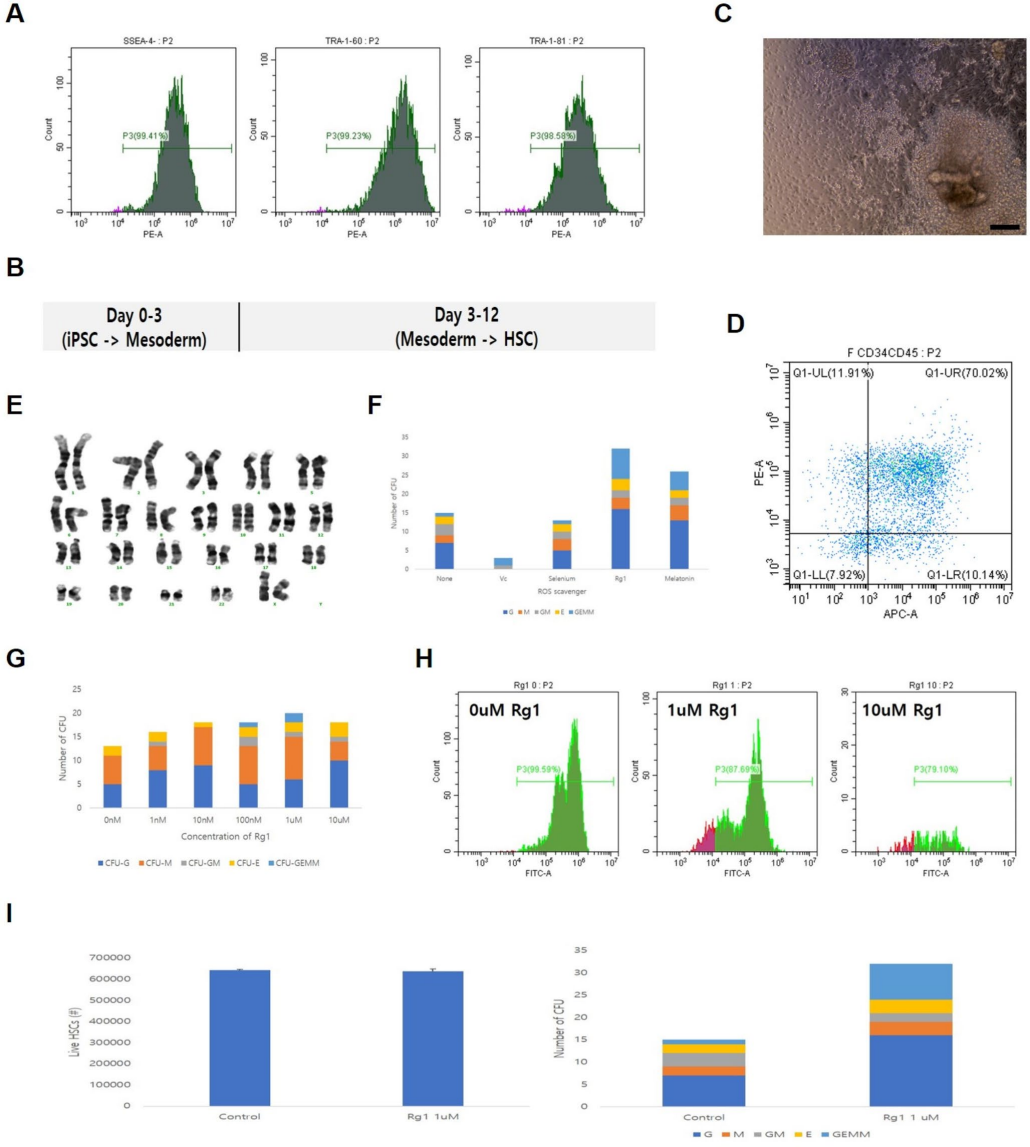
In vitro antifragile treatment for generation of high quality iHSC. High-quality iHSC can be produced by incorporating the antioxidant Rg1 during differentiation. **A** iPSC derived from dermal fibroblasts used in iHSC manufacturing demonstrate high pluripotency marker expression. **B** Differentiation of iPSC into iHSC proceeds in two stages (mesodermal specification and HSC differentiation) over a period of 12 days. **C** iHSC differentiation is similar to the in vivo differentiation process by forming a HSC suspension through budding in an attached HE. **D** iHSC expresses HSC markers CD34 and CD45 simultaneously. **E** iHSC maintains a normal karyotype. **F** Screening for diverse antioxidants during iHSC differentiation demonstrates that the best effects occur when Rg1 is incorporated. **G** 1 µM of Rg1 was incorporated during iHSC differentiation, demonstrating the best effect. **H** During iHSC differentiation, incorporating 1 µM Rg1 results in a decrease in ROS levels, while 10 µM Rg1 demonstrates a toxic effect. **I** 1 µM Rg1 induces qualitative improvement in iHSC. Scale bar = 200 μm. Results in (**A**), (**C**), (**D**), and (**H**) is representative image of three independent experiments. Results in (**I**) represent means ± SEM of three independent experiment. ^#^*p* < 0.01 and ^*^p < 0.05

During differentiation, high-quality iHSC can be produced in vitro by incorporating antioxidants. iHSC were produced by differentiating iPSC for 12 days, during which diverse pluripotency markers were expressed at 90% or higher (Fig. 2A-B). In vitro differentiation protocol comprised one stage (3 days) that was specified from iPSC to mesoderm, and two stages (9 days) that included differentiation from mesoderm to HSC (Fig. 2B). Similar to in vivo, in vitro suspended iHSC were produced by budding in the attached HE from day 9 of differentiation (Fig. 2C). More than 70% of the iHSC produced were CD34^+^CD45^+^ double-positive and maintained a normal karyotype (Fig. 2D-E). After treating diverse antioxidants (50 µg/ml ascorbic acid, 50 ng/ml selenium, 1 µM Rg1, and 10 µM melatonin) during iHSC differentiation, antioxidant efficacy was evaluated by the total number of colonies formed through the CFU assay. Rg1 exhibited the highest efficacy (Fig. 2F). When comparing Rg1 treatment concentrations, iHSC produced by incorporating 1 µM formed the largest number of total colonies (Fig. 2G). ROS generated during iHSC differentiation decreased due to Rg1 1 µM treatment, and a toxic effect was demonstrated at 10 µM (Fig. 2H). The beneficial effect of Rg1 was not a quantitative increase in the total number of iHSC, but a qualitative increase in iHSC that maintained their CFU-forming ability (Fig. 2I).

### In vivo Engraftment of iHSC after Strong Busulfan Conditioning

**Fig. 3:**
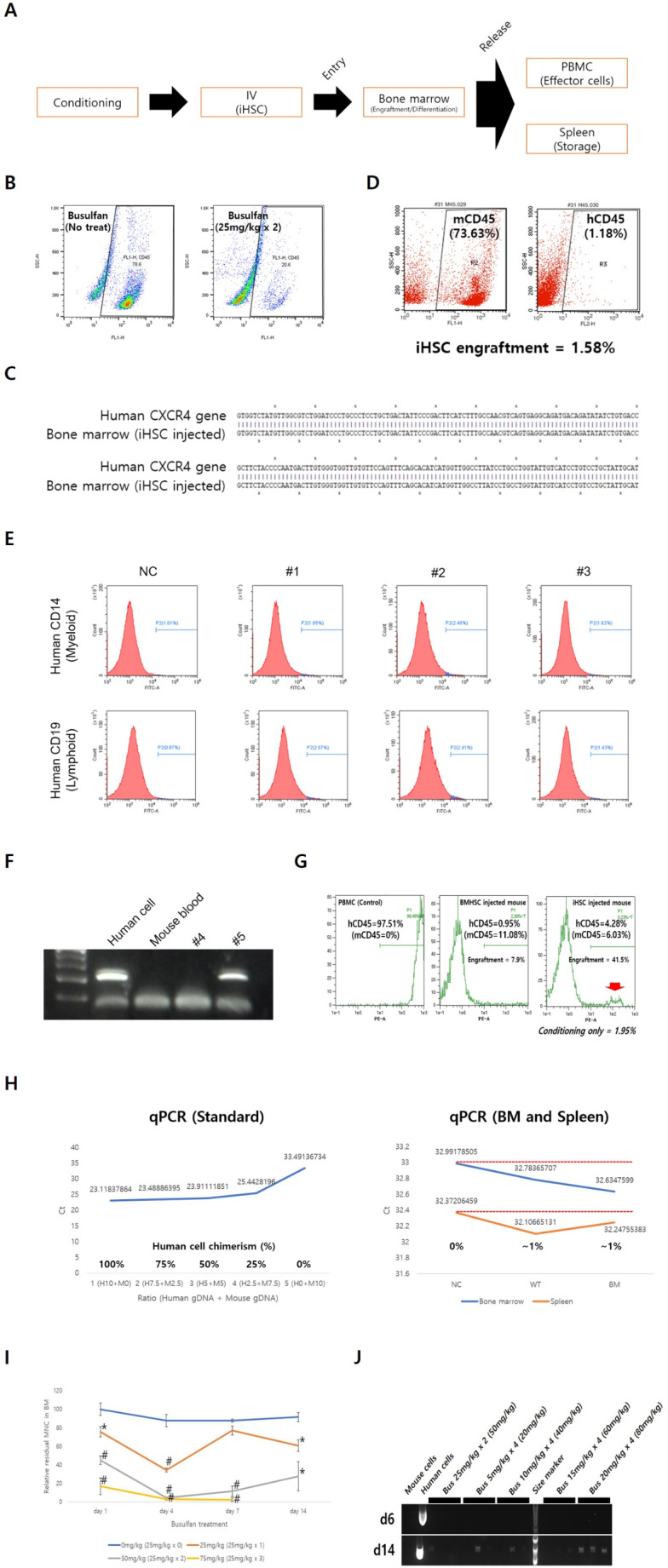
In vivo antifragile treatment for efficient engraftment of iHSC. Efficient iHSC engraftment is induced by strengthening busulfan conditioning. **A** Schematic depicting the process of iHSC entry into the BM, engraftment, differentiation, and release. **B** The number of mouse CD45^+^ cells in the BM are reduced when mice were conditioned with busulfan (50 mg/kg). **C** Human specific PCR is used to confirm iHSC entry in the BM after 8 week iHSCT. **D** Low engraftment observed post entry. **E** iHSC engrafted into the BM differentiated into myeloid (CD14^+^ monocytes) and lymphoid (CD19^+^ B cells) lineages. **F** Human cells identified in the PB on day 6 of iHSCT, when the busulfan dose was increased to 125 mg/kg (25 mg/kg × 5). However, all mice died within 2 weeks owing to high-dose busulfan toxicity. **G** Increasing the busulfan dose to 200 mg/kg (50 mg/kg × 4) increased the number of human CD45^+^ cells in the PB at week 8 of iHSCT. The chimerism rate of human cells was 41.5%. However, the mortality rate 8 weeks post iHSCT was very high (6/7, 85.7%). **H** Human cells detected in the BM and spleen (WT: iHSC injected, BM: BMHSC injected). **I** When the busulfan conditioning dose is 50 mg/kg (25 mg/kg × 2), the BM residual MNC is bounded from day 7. Treatment with 75 mg/kg busulfan conditioning dramatically increased mortality. **J** Conditioning with a cumulative dose of 80 mg/kg (20 mg/kg × 4) and a 20% lower dose of busulfan increases short-term engraftment efficiency in week 2. Results in (**B**), (**D**), and (**E**) is representative image of three independent experiments. Results in (**I**) represent means ± SEM of 3 independent experiment. Results in (**J**) represent n = 3 mice per group. ^#^*p* < 0.01 and ^*^p < 0.05

Generally, immediately after HSC are transplanted through IV into conditioned mice, the cells migrate to the BM for entry / engraftment, HSC-derived differentiated cells migrate to the PB, and excess differentiated cells are stored in the spleen (Fig. 3A). A humanized mouse is generally recognized while more than 25% of human CD45^+^ cells are detected in the PB. Immediately after busulfan conditioning was performed at 50 mg/kg (25 mg/kg × 2) in nude mice, it was verified that mouse CD45^+^ cells were reduced by more than 70% in the BM (Fig. 3B). Although busulfan concentrations exceeded 50 mg/kg, most mice died of toxicity (Table 2). After conditioning the mice with busulfan at 50 mg/kg, iHSC entered the BM qualitatively by IV injection (Fig. 3C). However, a low engraftment rate was observed (Fig. 3D). The engrafted cells differentiated in vivo into myeloid and lymphoid lineage cells (Fig. 3E). Immediately after the busulfan dose was increased to 125 mg/kg (25 mg/kg × 5) during conditioning, direct PCR verified that a large number of human cells were present in the PB on the 6th day post iHSCT (Fig. 3F). However, the mice died on day 13 due to high dose busulfan toxicity (Fig. 3F; Table 2). When the busulfan dose was increased to 200 mg/kg (50 mg/kg × 4), mice with up to 41.5% human cell chimerism in the PB were observed (Fig. 3G; Table 2). Human cells were also detected in the BM and spleen (Fig. 3H). However, mortality was extremely high (85.7%, 6/7; Table 2). As the busulfan dose increased, the number of MNC in the BM decreased (Fig. 3I). A commonly used busulfan dose of 50 mg/kg (25 mg/kg × 2) was administered, and a decreasing pattern was observed until day 4; however, post day 7, the MNC remaining in the BM recovered (Fig. 3I). At busulfan dose of 75 mg/kg (25 mg/kg × 3), all mice died within 8 weeks (Table 2). To lower mortality, the busulfan dose was reduced by 20% compared to the previous dose (25 mg/kg to 20 mg/kg) and the cumulative dose was adjusted to 80 mg/kg with four doses, mortality decreased (33.3% survival at 8 weeks) and short-term engraftment efficiency at 2 weeks increased (100%, 3/3) (Table 2; Fig. 3J).

**Table 2:** Comparison of in vivo engraftment and mortality by Busulfan cumulative dose

Conditioning		Human cell chimerism		Mortality rate at 8 wks (/head)
Busulfan cumulative dose	Protocol (one dose x number of times)	Short-term engraftment (PCR at 2 wks) (/ head)	Long-term engraftment (FACS at 8 wks) (/ MNC)	
0 mg/kg	-	0% (0/2)	N/A^$^	0% (0/2)
20 mg/kg	5 mg/kg x 4	33.3% (1/3)	0%^*^	0% (0/3)
25 mg/kg	25 mg/kg x 1	0% (0/7)	N/A^$^	14.3% (1/7)
40 mg/kg	10 mg/kg x 4	33.3% (1/3)	0%^*^	0% (0/3)
50 mg/kg	25 mg/kg x 2	6.5% (5/77)	1.63 ± 0.79%^*^	46.8% (36/77)
60 mg/kg	15 mg/kg x 4	0% (0/3)	N/A^$^	0% (0/3)
75 mg/kg	25 mg/kg x 3	0% (0/4)	N/A^$^	100% (4/4)
80 mg/kg	20 mg/kg x 4	100% (3/3)	0%^*^	66.7% (2/3)
100 mg/kg	25 mg/kg x 4	0% (0/2)	N/A^$^	100% (2/2)
125 mg/kg	25 mg/kg x 5	50% (1/2)^#^	N/A^$^	100% (11/11)
200 mg/kg	50 mg/kg x 4	N/A^$^	41.5%^*^	85.7% (6/7)

^*^Human cell chimerism (%) = [hCD45 (%) / (hCD45 (%) + mCD45 (%)) x 100]_experimental group_ - [hCD45 (%) / (hCD45 (%) + mCD45 (%)) x 100]_control group_

^#^Analysis on day 6 of iHSCT. Mortality rate on day 6 is 9/11 (81.8%)

^$^N/A, Not applicable

## Discussion

In this study, two antifragile treatments were verified to enable high-quality iHSC to be produced in vitro and efficiently engrafted into the BM in vivo. When differentiating iPSC into HSC in vitro, the antioxidant Rg1 was incorporated at a concentration of 1 µM, and the ROS level during differentiation decreased, which increased the number of colony-forming cells. When mice were conditioned with busulfan 50 mg/kg before implantation, entry and engraftment into the BM of iHSC and lymphoid/myeloid lineage differentiation had low efficiency but were still feasible. With the increase in busulfan dose to 125 or 200 mg/kg, the engraftment efficiency of iHSC increased, but mortality also increased. To overcome this problem, it was established that when the total dose was increased by lowering the single busulfan dose and increasing the frequency, iHSC engraftment increased and the mortality rate decreased. This study demonstrated that the entire engraftment process (BM entry, engraftment, lymphoid/myeloid differentiation, release, and storage) of iHSC is possible with high efficiency.

Despite the active clinical research using diverse differentiated cells derived from pluripotent stem cells (PSC) (embryonic stem cells (ESC) and iPSC), this is not true for iHSC [17]. Differentiated iHSC demonstrate a phenomenon, wherein a HSC-specific marker is properly expressed; however, in vivo engraftment is rarely performed [18]. Cells (HSC) and organs (spinal cord and brain) typically located in bones are very “fragile”. HSC easily lose their multipotency during in vitro culture; therefore, they are transplanted immediately after collection without a culture process. Since the process of differentiating iPSC into HSC in vitro takes approximately two weeks, the transplanted iHSC must suffer significant damage compared to freshly collected blood-derived HSC. Therefore, for iHSC to be used for therapeutic purposes, additional “antifragile treatment” conditions must be added to existing differentiation conditions. ROS is a major cause of damage in HSC differentiation, and several studies have reported that ginsenoside Rg1 helps maintain HSC quality [10, 11]. In this study, Rg1 was introduced into the iHSC production process to reduce the ROS generated during differentiation. As a result, high-quality iHSC with increased colony-forming cell numbers could be produced.

It was verified that iHSC engraftment increased when the busulfan dose was increased from the commonly used 50 mg/kg to 125 or 200 mg/kg during conditioning. Interestingly, when busulfan (Busulfex^®^) administration doses widely used in human HSCT, 12.8 mg/kg (adult standard) and 17.6 mg/kg (children below 12 kg standard) were converted into animal doses (mouse), they were 157.44 mg/kg (adult standard) and 216.48 mg/kg (pediatric standard), which is quite similar to the dose used to obtain meaningful engraftment results in this study (125 and 200 mg/kg). In general, mice are treated with busulfan at a concentration of 50–80 mg/kg, which appears to be a set dose owing to the problem of rapidly increasing mortality when the dose was increased. When the dosages were similarly matched (75–> 80 mg/kg) by lowering the first busulfan dose by 20% (25–> 20 mg/kg) and increasing the frequency (3–> 4), it was verified that mortality at week 8 decreased (100–> 66.7%) and the short-term engraftment ratio at week 2 increased (0–> 100%) (Table 2; Fig. 3J). Busulfan at 80 mg/kg was not the optimal dose for iHSC engraftment; therefore, iHSC seems to be limited to short-term engraftment, and the optimal busulfan dose is 125 mg/kg or more (Table 2).

In summary, the following conditions must be met for iHSC to engraft into the BM with high efficiency: First, sufficient empty space must be provided in the BM during iHSCT to ensure smooth BM entry. To do so, sufficient busulfan administration of 125 mg/kg or more is essential, and it seems that sufficient space for iHSC to enter the BM is created only 4–5 days post the first conditioning (Table 2; Fig. 3I). Second, following entry, iHSC need be firmly engrafted into the BM. A static BM niche that effectively inhibits residual BM cell proliferation must be maintained during the engraftment process. Busulfan seems to inhibit BM residual cell proliferation for approximately four days post administration; implanting iHSC on the first day post last conditioning will sufficiently inhibit BM residual cell proliferation until engraftment is completed. Third, the increasing mortality associated with increasing busulfan levels should be reduced. This was resolved by administering a low busulfan concentration (≤ 20 mg/kg) in multiple doses. Collectively, a protocol that divides busulfan into small amounts of less than 20 mg/kg and administers it over a period of 4 days to adjust the cumulative dose to 125 mg/kg or more, followed by implanting iHSC on the first day of the last busulfan administration, seems to be the best protocol for engraftment.

To date, the highest in vivo engraftment report with PSC-derived HSC, wherein 27.9% of human CD45^+^ cells were detected in mouse PB after transplanting human ESC-derived HSC [19]. In this report, mouse feeder cells were used for HSC differentiation and high engraftment rate was achieved by intrafemoral injection of HSC into total-body irradiation-conditioned NOD scid gamma (NSG) mice. Herein, iHSC were manufactured using a feeder-free method and intravenously injected into busulfan-conditioned nude mice; 41.5% of human cell chimerism was confirmed based on human CD45^+^ cells in the PB. The phenomenon of a significantly lower engraftment rate in BM than in PB is similar to previous studies [19]. Also, the low human cell chimerism observed in the spleen is thought to be due to the analysis after isolating the spleen from recipient mice and separating DNA without human cell sorting.

This study demonstrated that high-efficiency engraftment is possible by producing HSC using only morphogens. Results that increases the engraftment efficiency of iHSC by non-genetically is significant in terms of clinical translation. This is because the random integration of the expression vector can be a cause of threatening the safety of the drug [20]. In addition, a high engraftment level was obtained using only T cell-deleted Balb/c-nude mice. Previous related study used fully immunodeficient mice (NSG) with simultaneous depletions of NK cells and macrophages as well as T cells as recipient mice [19]. It is noteworthy that high engraftment efficiency was achieved even with unfavorable recipients compared to previous related studies [19]. Better engraftment results are expected in the future, if research is performed using strongly immunodeficient mice.

The biggest significance of this study is the finding of clues regarding high efficiency iHSC engraftment. Further optimization is expected to yield better results. There may be a variety of antioxidants that can more effectively control ROS levels, which were found to be the major damaging factors. Consequently, an antioxidant cocktail may be useful for iHSC differentiation. It is essential to find an efficient approach that does not increase mortality even when the busulfan dose is increased. Thus, it is essential to screen diverse administration pathways, such as low-dose / long-term treatment method, which was explored primarily in this study (Table 2; Fig. 3J) or direct injection of busulfan into the BM. Thus, it will be possible to verify the high efficiency iHSC engraftment conditions more precisely.

In conclusion, fragile iHSC are vulnerable to damage. iHSC do not engraft well as they are easily damaged during in vitro differentiation and in vivo engraftment. For iHSC to be engrafted in vivo with high efficiency, it is essential to remove as many damaging factors as possible during differentiation to create high-quality iHSC and a BM niche that is favorable for engraftment when iHSC enter the in vivo environment. Herein, Rg1 treatment during the in vitro differentiation process was sufficient to produce iHSC capable of proceeding with a low-efficiency engraftment process. Additionally, highly efficient in vivo iHSC engraftment conditions were found by fine-tuning the busulfan conditioning conditions. In the future, iHSC will be considered a patient-specific treatment for blood cancer, autoimmune diseases, and infectious diseases [21].

## Data Availability

The data that support the findings of this study are available from the corresponding author on reasonable request.

## Abbreviations

BM: Bone marrow
CFU: Colony forming unit
DCFDA: 2` 7`-dichlorofluorescin diacetate
ESC: Embryonic stem cells
HE: Hemogenic endothelium
HSC: Hematopoietic stem cells
HSCT: Hematopoietic stem cells transplantation
iHSC: Induced pluripotent stem cells derived hematopoietic stem cells
iHSCT: Induced pluripotent stem cells derived hematopoietic stem cells transplantation
IP: Intraperitoneal
iPSC: Induced pluripotent stem cells
IV: Intravenous
LT-HSC: Long-term repopulating hematopoietic stem cells
MNC: Mononuclear cells
N/A: Not applicable
NK: Natural killer
NSG: NOD scid gamma
PB: Peripheral blood
PBMC: Peripheral blood mononuclear cells
PSC: Pluripotent stem cells
ROS: Reactive oxygen species
TCP: Tissue culture plate
